# MiR-155 Negatively Regulates Anti-Viral Innate Responses among HIV-Infected Progressors

**DOI:** 10.3390/v15112206

**Published:** 2023-11-01

**Authors:** Puja Pawar, Jyotsna Gokavi, Shilpa Wakhare, Rajani Bagul, Ujjwala Ghule, Ishrat Khan, Varada Ganu, Anupam Mukherjee, Ashwini Shete, Amrita Rao, Vandana Saxena

**Affiliations:** 1 Division of Immunology and Serology, ICMR-National AIDS Research Institute, Pune 411026, India; pujappawar96@gmail.com (P.P.); jyotsgokavi@gmail.com (J.G.); vwakhare2@gmail.com (S.W.); varada.ganu@gmail.com (V.G.); ashete@nariindia.org (A.S.); 2 Division of Clinical Sciences, ICMR-National AIDS Research Institute, Pune 411026, India; rajanibagul@yahoo.com (R.B.); gawadeujjwala5@gmail.com (U.G.); arao@nariindia.org (A.R.); 3 Division of Virology, ICMR-National AIDS Research Institute, Pune 411026, India; ishratkhan09@gmail.com (I.K.); amukherjee@nariindia.org (A.M.)

**Keywords:** HIV, disease progression, LTNPs, TLRs, innate, miR-155, host restriction factors

## Abstract

HIV infection impairs host immunity, leading to progressive disease. An anti-retroviral treatment efficiently controls viremia but cannot completely restore the immune dysfunction in HIV-infected individuals. Both host and viral factors determine the rate of disease progression. Among the host factors, innate immunity plays a critical role; however, the mechanism(s) associated with dysfunctional innate responses are poorly understood among HIV disease progressors, which was investigated here. The gene expression profiles of TLRs and innate cytokines in HIV-infected (LTNPs and progressors) and HIV-uninfected individuals were examined. Since the progressors showed a dysregulated TLR-mediated innate response, we investigated the role of TLR agonists in restoring the innate functions of the progressors. The stimulation of PBMCs with TLR3 agonist-poly:(I:C), TLR7 agonist-GS-9620 and TLR9 agonist-ODN 2216 resulted in an increased expression of IFN-α, IFN-β and IL-6. Interestingly, the expression of *IFITM3*, *BST-2*, *IFITM-3*, *IFI-16* was also increased upon stimulation with TLR3 and TLR7 agonists, respectively. To further understand the molecular mechanism involved, the role of miR-155 was explored. Increased miR-155 expression was noted among the progressors. MiR-155 inhibition upregulated the expression of TLR3, NF-κB, IRF-3, TNF-α and the *APOBEC-3G*, *IFITM-3*, *IFI-16* and *BST-2* genes in the PBMCs of the progressors. To conclude, miR-155 negatively regulates TLR-mediated cytokines as wel l as the expression of host restriction factors, which play an important role in mounting anti-HIV responses; hence, targeting miR-155 might be helpful in devising strategic approaches towards alleviating HIV disease progression.

## 1. Introduction

A human immunodeficiency virus type 1 (HIV-1) infection leads to immune dysregulation, resulting in rampant viral dissemination and progression to AIDS [1,2,3]. Among HIV-infected individuals, a loss of CD4 count and increased viral load are the hallmarks of a progressive disease [4,5,6]. Anti-retroviral treatment (ART) regimens greatly extend the survival time of HIV-infected persons by controlling the viremia, but it cannot eliminate the latent reservoirs [7,8,9]. Furthermore, it fails to reverse the HIV-induced immune dysfunction [10,11,12,13], which renders such individuals vulnerable to not only acquiring other opportunistic infections but also results in faster disease progression in cases of treatment interruption or failure [14,15]. Therefore, it is very important to understand the factors associated with immune dysfunction contributing to rapid disease progression, which will help in the development of new immunotherapeutic interventions or strategies to restore immune functions. While adaptive immune dysfunction has been widely studied [16,17,18], much less is known about the role and regulation of innate immune mechanisms during HIV disease progression. 

Besides being the first line of antiviral defence, innate immunity plays a critical role not only in restricting the establishment of HIV-1 infection due to anti-viral cytokines such as type-1 IFN and proinflammatory cytokines [19,20,21], but also in activating the effector immune functions of adaptive immune system [22,23]. Type-1 IFNs, in turn, lead to the upregulation of interferon-stimulated genes (ISGs) such as *IFITM*, *BST-2*, *APOBEC*, *SAMHD1*, *Mx10*, *IP-10*, *BST/tetherin*, *TRIM5*, *GBP5*, *ZAP*, *CNP*, *Mov10*, etc., family members that have been found to inhibit HIV infection [24,25,26]. In this way, innate immunity is vital in maintaining an overall antiviral immune environment in host cells. Furthermore, increasing evidence suggests that innate immune responses are also crucial in shaping the disease outcome post infection [27,28]. Innate signalling pathways are triggered following receptor–ligand interactions, which involve the engagement of distinct classes of pattern recognition receptors (PRRs) such as Toll-like receptors (TLRs), RIG-I-like receptors (RLRs), Nod-like receptors (NLRs), AIM2-like receptors (ALRs), C-type lectin receptors (CLRs) and cGAS [29,30]. Among these, TLR-mediated responses have been largely studied and shown to play a role in HIV immunopathogenesis as well [31,32,33,34]. While TLR2 and TLR8 have been shown to promote virus replication and immune activation [35,36,37,38], the stimulation of TLR4, TLR3, TLR7/8 and TLR9 inhibited HIV replication in various cells such as primary monocyte-derived macrophages (MDM), monocytes and PBMCs [39,40,41,42,43,44,45]. Further, TLR7 and TLR8 play important roles in sensing HIV-1 in pDCs and CD4+ T cells [46,47]. TLR8 stimulation improves T cell receptor signalling and increases cytokine secretion in HIV-infected CD4+ T cells [47]. Additionally, the differential role of TLRs has been associated with different disease outcomes [48,49]. Being dynamic in nature, a HIV infection results in rapid disease progression, leading to AIDS in the absence of ART; however, a small population of infected individuals do not progress to severe illness despite being ART-naïve. These asymptomatic individuals are long-term non-progressors who maintain a stable CD4 count [50]. Evidences suggest that LTNPs have better immune functions than those of progressors [51,52,53,54]. A transcriptome analysis by Wu et al. highlighted that HIV-infected progressors had an overall down-regulation of the TLR signalling pathway, along with lower MAPK and NF-ĸB activation [55]. Further, reduced expressions of TLR7/8 and TNF-α secretion were reported in chronic and AIDS subjects compared to LTNPs [42]. These studies collectively suggest that TLR-mediated innate immunity differentially plays a role during HIV immunopathogenesis and may also influence the disease outcome. However, most of the available data regarding the role of TLR-mediated innate immune responses and its association with reduced viral growth and/or disease progression have been gathered through cohort studies, in vitro studies or ex vivo studies (where HIV infection was established in the cells obtained from healthy donors) [44,56,57,58]. Given that HIV infection escapes the host immune response by impairing the functional immune mechanism(s), including innate immunity, in the infected individuals [59,60], none of the available studies provide enough evidence that the modulation of innate immune signalling can help in ameliorating the anti-viral innate immune responses in such individuals. Hence, this study was conducted to examine whether augmenting TLR-mediated immune signalling helps in restoring the innate immune functions in the cells of HIV-infected progressors. We further wanted to understand the molecular mechanism involved in the regulation of innate immunity in the progressors. In addition to other protein factors, microRNAs (miRNAs/miRs) have emerged as one of the important mediators of immunity [61]. MiRNAs have been shown to inhibit HIV-1 expression either by modulating the host innate immunity or directly by interfering with viral mRNAs [62], indicating their role in HIV immunopathogenesis. MiR-155, in particular, has been shown to not only play a crucial role in regulating innate and antiviral responses in various viral infections [63,64] but has also been identified as a potential biomarker of HIV-induced immune dysfunction [65]; however, its role in regulating innate immune responses is not well defined, which was examined in the current study.

In this study, a compromised innate immune response was observed during HIV disease progression, where HIV-infected progressors had a reduced expression of endosomal TLRs and downstream innate immune components such as type-1 interferon (IFN), innate cytokines and IFN-induced anti-viral ISGs than that of LTNPs. Stimulation with the TLR3, TLR7 and TLR9 agonists helped improve the innate cytokine response in the progressors. The progressors also had an increased expression of miR-155, and the inhibition of miR-155 augmented the expression of innate cytokines as well as host restriction factors such as *APOBEC-3G, IFI-16, IFITM-3* and *BST-2*.

## 2. Materials and Methods

### 2.1. Study Samples

In this study, 25 (9 M/16 F) long-term non-progressors (LTNPs), 37 (17 M/20 F) progressors and 33 (21 M/12 F) HIV-uninfected individuals were enrolled at the ICMR—National AIDS Research Institute Clinic at Pune, India. The median age was 39 years for LTNPs (range 19–54 years), 42 years for progressors (range 28–55 years) and 32 years for HIV-uninfected individuals (range 20–52 years). The median seropositivity period of LTNPs was 11 years (range 8–19 years). Progressors were defined as anti-retroviral treatment (ART)-naïve HIV-infected patients with a CD4 count between 300 and 500 cells/mm^3^, while LTNPs were asymptomatic HIV-infected individuals who were ART-naïve with a stable CD4 count of ≥500 cells for 7 or more years [66]. The study was approved by the institutional ethics committee and the study participants were enrolled in the study after obtaining their written informed consent. From the study participants, whole blood samples were collected, followed by the isolation of peripheral blood mononuclear cells (PBMCs) and plasma using Ficoll hypaque density gradient centrifugation. PBMCs were cryopreserved in FBS with 10% dimethyl sulfoxide in liquid nitrogen, while plasma was stored at −80 °C until further use. 

### 2.2. CD4+ T Cell Counts and HIV Viral Load Estimation

CD4+ T cell counts (cells/mm^3^) were estimated in the whole blood samples of HIV-infected individuals by flow cytometry (FACSCalibur, Becton-Dickinson, San Jose, CA, USA) using a TruCOUNT kit (Becton-Dickinson, CA, USA) following the manufacturer’s instructions. In the plasma samples, HIV-1 viral load (RNA copies/mL) was assessed using Abbott m2000rt HIV-1 real-time PCR according to the manufacturer’s instructions. The lower limit of detection was 150 HIV RNA copies/mL. For the statistical analysis, values less than 150 RNA copies/mL were considered to be 150 RNA copies/mL.

### 2.3. Extraction of Total RNA, cDNA Synthesis and Quantitative Real-Time PCR (q-PCR) 

The frozen PBMCs from the study participants were revived and exhibited 80–90% viability, assessed using the trypan blue exclusion method. RNA isolation was performed using a Trizol reagent (Cat # AM9738, Invitrogen, Thermo Fisher Scientific, Waltham, MA, USA) followed by complementary DNA (cDNA) synthesis (Cat#RR037B, Takara Bio Inc., Kusatsu, Shiga, Japan) from 200 ng RNA, as per the manufacturer’s instructions. A PowerUp™ SYBR™ Green Master Mix (Cat#A25742, Applied Biosystems™, Thermo Fisher Scientific, Waltham, MA, USA) was used for a quantitative 7500 Fast Real-Time PCR System (Applied Biosystems™, Thermo Fisher Scientific, Waltham, MA, USA) to assess the expression of the various genes involved in innate immune pathways in HIV-infected and uninfected samples.

### 2.4. Stimulation of PBMCs with TLR agonists

Cryopreserved PBMCs isolated from progressors (*n* = 11) were revived in complete RPMI 1640 medium (Cat#31800022, Gibco^TM^) supplemented with 10% heat-inactivated fetal bovine serum (FBS) at 37 °C and 5% CO_2_. Depending on the cell availability, 50,000–80,000 cells were stimulated with various TLR agonists, i.e., TLR3 agonist—poly (I:C) (1 μm; Cat#tlrl-pic, Invivogen, San Diego, CA, USA), TLR7 agonist—GS-9620 (1 μm; Cat# HY-15601, MedChem Express, Monmouth Junction, NJ, USA) and TLR9 agonist—ODN-2216 (3 µm; Cat#TLRL-2216-1, Invivogen, San Diego, CA, USA). After 18 h, cells were harvested and used for real-time PCR assay to assess the expression of components of innate immune pathways, i.e., (i) transcription factors (NF-κB and IRF3); (ii) type-I interferons (IFN-α and IFN-β); and (iii) proinflammatory cytokines (TNF-α, IL-6, IL-1β). We also examined the effect of TLR agonist stimulation on the expression profiles of host restriction factor genes—*IFITM1*, *IFITM3*, *APOBEC-3G*, *IFI16* and *BST-2*. The primer sequences were customized from Integrated DNA Technologies, Coralville, IA, USA, as mentioned previously (referred in Table 1). 

### 2.5. Transfection of miR-155 Inhibitor in the PBMCs

50,000–80,000 PBMCs of progressors were transfected with a 200 nM inhibitor of mir-155-5p (Cat#IH-300647- 06-0020, Dharmacon, Lafayette, CO, USA) or a 200 nM scrambled mir inhibitor (negative control) (Cat#IN-001005-01-20, Dharmacon, Lafayette, CO, USA) using the Qiagen HiPerfect kit transfection protocol (Cat#301704, Hilden, Germany), as directed by the manufacturer. After 24 h of incubation at 37 °C with 5% CO_2_incubator, PBMCs were washed three times with phosphate-buffered saline (PBS) and cells were harvested in Trizol reagent followed by RNA extraction. For miRNA quantification, a TaqMan™ MicroRNA Reverse Transcription Kit (Cat#4366596, Applied Biosystems™, Thermo Fisher Scientific, Waltham, MA, USA) was used for cDNA synthesis, and mir-155 inhibition was determined by RT-PCR via miR-155 and RNU-44TaqMan™ MicroRNA assays. TaqMan™ Universal Master Mix-II (Cat#4440040 Applied Biosystems™, Waltham, MA, USA) and TaqMan™ primer-probes for miR-155 and RNU-44 were used to perform real-time PCR.

To determine the expression of TLRs, transcription factors, host restriction factors, type-1 interferons and innate immune cytokines, cDNA was synthesized from 100 ng of total RNA, extracted using the PrimeScript RT reagent Kit (Cat#RR037B, Takara Bio Inc., Kusatsu, Shiga, Japan), and a RT-PCR was performed using the PowerUp™ SYBR™ Green Master Mix (Cat#A25742, Applied Biosystems™, Thermo Fisher Scientific, Waltham, MA, USA) using specific primers (Table 1). The relative gene expression and fold change graph was plotted, which was normalized to β-actin, and miR-155 expression was normalized to RNU44. The fold change was calculated using the 2^−ΔΔCt^ method.

Flow cytometry was performed to detect the intracellular expression of TNF-α and IFN-α proteins in the PBMCs of progressors post transfection with miR-155 inhibitor and scrambled miR-inhibitor (mock). Briefly, cells were harvested post transfection, and standard intracellular cytokine staining was performed using anti-TNF-α-FITC and anti-IFN-α-AlexaFluor647 antibodies (BD Biosciences, Franklin Lakes, NJ, USA). The Fixable Aqua dead cell stain kit (Invitrogen, Waltham, MA, USA) was used to gate the live population. Fluorescence minus one (FMO) was used as a control for the gating strategy (Supplementary Figure S1). The cells were acquired and analysed on FACSAria Fusion (Becton Dickinson, Franklin Lakes, NJ, USA) using FlowJo software Version 10.0.

### 2.6. miRNA-mRNA Target Prediction

Bioinformatics tools were used to predict multiple potential binding sites of miRNAs in large target RNAs. To predict the miRNA–mRNA interactions, the 3′ UTR sequences of the genes viz. NF-κB, IRF-3, *APOBEC-3G, IFITM-3*, *IFI-16* and *BST-2* were obtained from the UCSC browser genome build GRCh38 (https://genome.ucsc.edu/; accessed on 24 March 2023) [83], and miRBase (https://www.mirbase.org/; accessed on 24 March 2023) was used to extract the miR-155 sequence [84]. The RNA hybrid server (https://bibiserv.cebitec.uni-bielefeld.de/rnahybrid; accessed on 24 March 2023) was used to find the energetically most favourable hybridization sites of miR-155 in a target mRNA [85,86].

### 2.7. Statistics

GraphPad Prism version 8.0 was used for the statistical analysis. The Mann–Whitney test was used to compare the mean levels of expression between different study groups, while the Wilcoxon matched-pairs signed ranks test was used for the paired sample analysis. A *p* value of ≤0.05 was considered to be significant. To assess the correlation between different parameters, the Spearman rank correlation test was used.

## 3. Results

### 3.1. Altered Innate Immune Responses during HIV Infection

**Figure 1:**
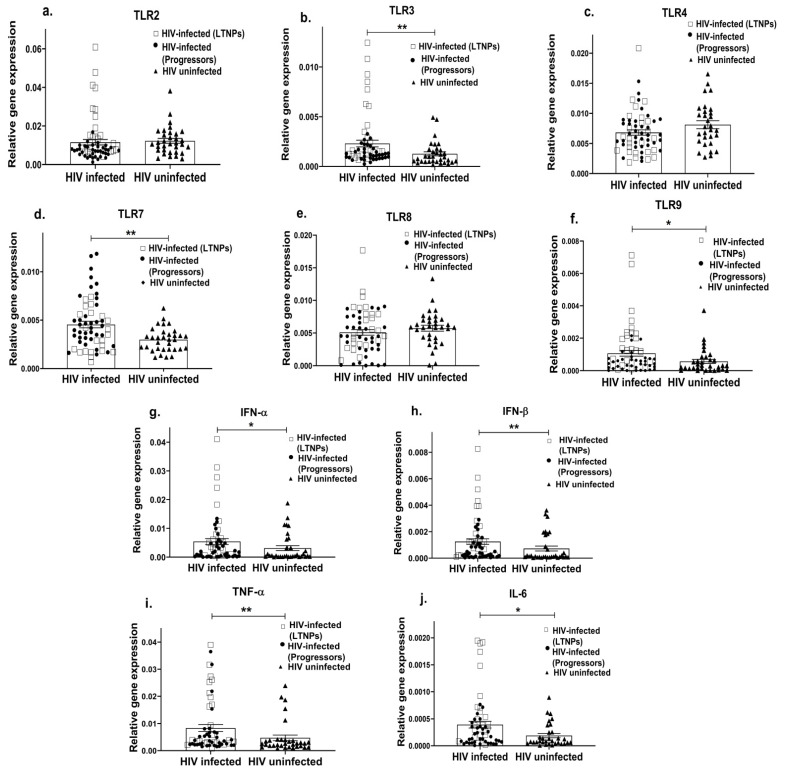
Altered innate immune response in HIV-infected individuals. PBMCs from HIV-infected (including LTNPs shown as squares and progressors shown as filled circles) and HIV-uninfected individuals (shown as filled triangles) were used to assess the gene expression profile of various TLRs such as TLR2 (**a**), TLR3 (**b**), TLR4 (**c**), TLR7 (**d**), TLR8 (**e**), TLR9 (**f**) and innate immune cytokines such as IFN-α (**g**), IFN-β (**h**), TNF α (**i**), IL-6 (**j**). The relative gene expression of these genes was normalized to β-actin. Statistical analysis was performed by using the Mann–Whitney test. * indicates *p* value < 0.05 and ** indicates *p* value < 0.01.

Since TLR-mediated innate immunity plays a crucial role in viral infections [87], we first examined the gene expression profile of the most common TLRs associated with viral infections, i.e., TLR-2, 3, 4, 7, 8, 9 in the PBMCs of HIV-infected (including both LTNPs and progressors) and HIV-uninfected individuals. We found that HIV-infected individuals had an upregulated expression of endosomal TLRs—TLR3 (*p* = 0.005; Figure 1b), TLR7 (*p* = 0.004; Figure 1d) and TLR9 (*p* = 0.02; Figure 1f), as compared to the HIV-uninfected individuals, while no significant difference was noted in the mRNA expression of TLR2 (*p* = 0.06; Figure 1a), TLR4 (*p* = 0.07; Figure 1c) and TLR8 (*p* = 0.23; Figure 1e). TLR signalling results in the expression of important antiviral cytokines, i.e., type-1 IFN (IFN-α and IFN-β) and proinflammatory cytokines (TNF-α, IL-6); we therefore assessed the expression profile of these innate cytokines. The HIV-infected individuals showed a higher expression of the IFN-α (*p* = 0.04; Figure 1g), IFN-β (*p* = 0.01; Figure 1h), TNF-α (*p* = 0.007; Figure 1i) and IL-6 (*p* = 0.02; Figure 1j) genes compared to the HIV-negative individuals. However, the HIV-infected group, which included both LTNPs and progressors, showed that the higher expression levels were more commonly expressed among the LTNPs cohort, as indicated in Figure 1. Considering that there may be variations between these two groups of HIV-infected individuals in terms of time of infection, suppression, etc., we therefore further compared these two groups individually.

### 3.2. Impaired Innate Immune Response Associated with HIV Disease Progression

**Figure 2:**
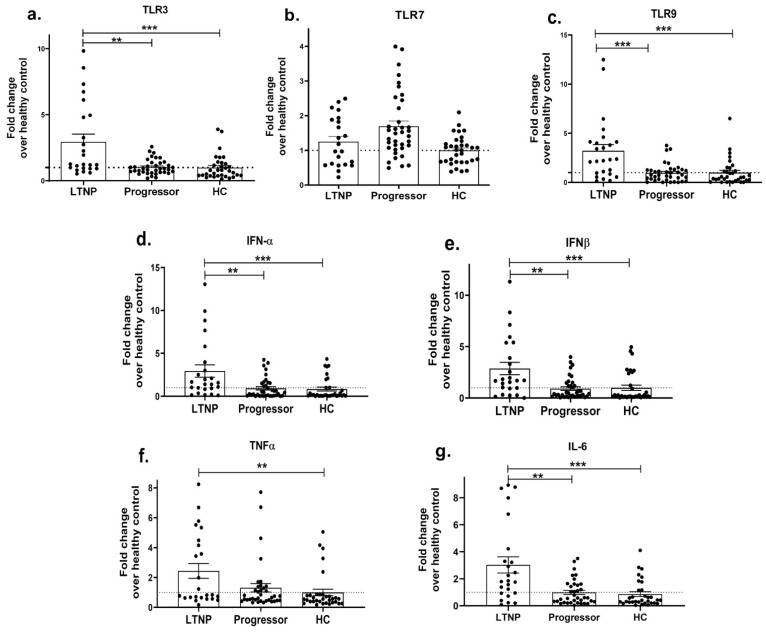
Impaired innate immune response during HIV disease progression. PBMCs isolated from LTNP and progressor study groups were used to analyse the gene expression of TLRs 3 (**a**), 7 (**b**) and 9 (**c**) and innate immune cytokines, i.e., IFN-α (**d**), IFN-β (**e**), TNF α (**f**), IL-6 (**g**). Fold change expression was calculated as 2^−ΔΔCt^ over the HIV-uninfected individuals, denoted as healthy control (HC), which is indicated as a dotted line. Statistical analysis was performed by using Mann–Whitney test. ** indicates *p* value < 0.01 and *** indicates *p* value < 0.001.

HIV infection progresses at differential rates in different individuals, where it may result in a progressive disease condition leading to AIDS or remain asymptomatic even in the absence of ART. Both viral factors and impaired host immune responses have been shown to contribute towards faster disease progression [88,89]. Since innate immunity is not only among the first events to mount but also shapes the adaptive immune response, we next questioned whether innate immunity had any association with HIV-progressive disease. To examine this, we first assessed the expression profile of endosomal TLRs, i.e., 3, 7, 9 (since these TLRs were significantly increased in the HIV-infected individuals, as shown in Figure 1) and innate cytokines in different cohorts of ART-naïve, HIV-infected individuals, i.e., progressors with a reduced CD4 count and long-term non-progressors (LTNP) who maintained a stable CD4 count for many years. The mRNA expressions of TLR3 (*p* = 0.001; Figure 2a) and TLR9 (*p* = 0.0002; Figure 2c) were found to be significantly downregulated in the progressors compared to the LTNPs, while no significant difference was noted in the expression of the TLR7 gene between the progressors and LTNPs (*p* > 0.05; Figure 2b). Further, the progressors showed a significant decrease in the expression levels of the IFN-α (*p* = 0.002; Figure 2d), IFN-β (*p* = 0.001; Figure 2e), IL-6 (*p* = 0.002; Figure 2f) and TNF-α (*p* = 0.053; Figure 2g) genes than the LTNPs.

**Figure 3:**
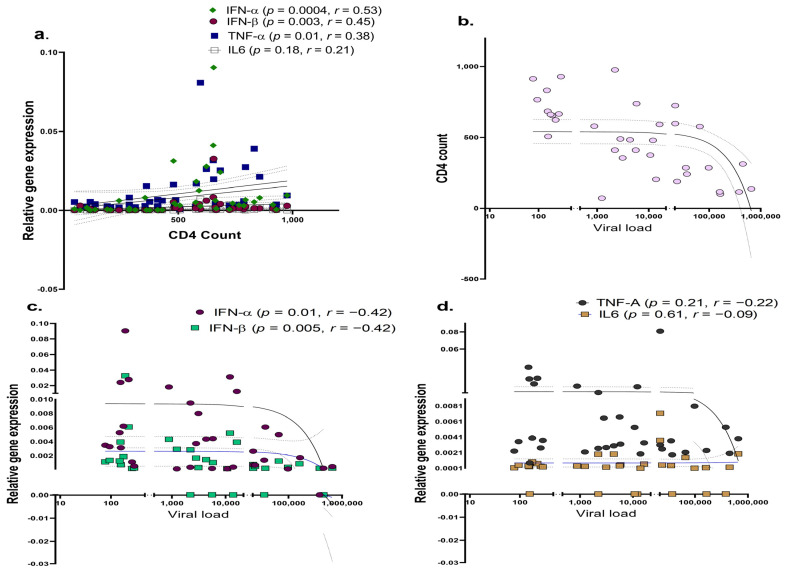
Correlation between innate immune cytokines and HIV-1 viral load/CD4 count in HIV-infected individuals. Correlation between IFN-α, IFN-β, TNF α and IL-6 mRNA levels with CD4 count (**a**), between CD4 count with viral load (**b**), between IFN-α, IFN-β mRNA levels with viral load (**c**) and between TNF α, IL-6 mRNA levels with viral load (**d**). The relative gene expression of these genes was normalized to β-actin. Correlation analysis was performed by using Spearman’s test.

We further determined the HIV-1 viral load (VL) and CD4+ T cell counts among the progressors and LTNPs, which are surrogate markers of disease progression. The progressors had a significantly higher viral load (mean = 121165; 1236–656,537 copies/mL) than the LTNPs (mean = 7853; 77–66,090 copies/mL) (*p* = 0.0002), while they also had a reduced range of CD4 count (median = 286; 46–489 cells/mm^3^) than those of the LTNPs (Median = 675; 500–976 cells/mm^3^), (*p* < 0.0001). Since we noted a reduced expression of innate cytokines in the progressors, we next sought to determine whether cytokine levels in HIV-infected individuals had any correlation with (i) HIV-1 viral load and (ii) CD4 count. The spearman correlation test showed that type-1 IFN mRNA levels—IFN-α and IFN-β, were positively correlated with CD4 count (*p* = 0.0004 and *p* = 0.003; Figure 3a; Table 2) and negatively correlated with HIV-1 viral load (*p* = 0.01 and *p* = 0.005; Figure 3c; Table 2). A higher level of TNF-α expression was found to be associated with an increased CD4 count (*p* = 0.01; Figure 3a; Table 2) but not with viral load (*p* = 0.21; Figure 3d; Table 2). No significant association was, however, noted between IL-6 levels and CD4 count or viral load (*p* = 0.18 and *p* = 0.61; Figure 3a,d; Table 2). Collectively, these results suggest that the suppressed expression of innate cytokines during HIV infection is associated with the HIV disease progression. 

### 3.3. Differential Expression of Host Restriction Factors in LTNPs and Progressors

**Figure 4:**
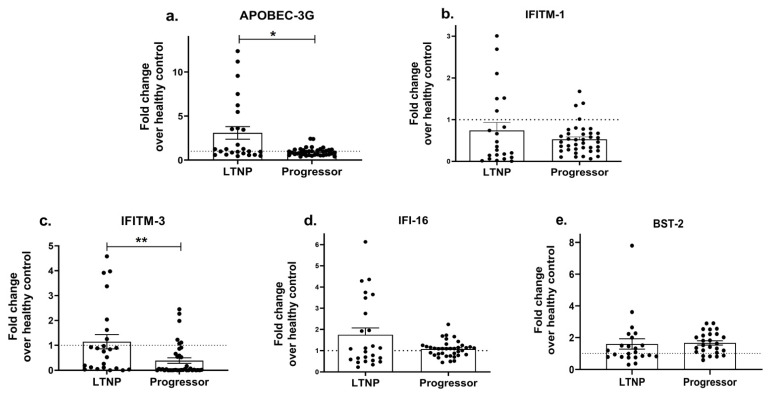
Differential expression of host restriction factors in LTNPs and progressors. PBMCs isolated from LTNPs and progressors study groups were used to analyse the gene expression of host restriction factors such as APOBEC-3G (**a**), IFTIM-1 (**b**), IFTIM-3 (**c**), IFI-16 (**d**) and BST-2 (**e**). Fold change expression was calculated as 2^−ΔΔCt^ over the HIV-uninfected individuals, denoted as healthy control (HC), which is indicated as a dotted line. Statistical analysis was performed by using Mann–Whitney test. * indicates *p* value < 0.05 and ** indicates *p* value < 0.01.

As host restriction factors play a significant role in regulating HIV disease progression and the type-I interferon-induced expression of interferon-stimulating genes (ISGs) [90] hampers viral growth, we next determined whether the expression profiles of ISGs such as APOBEC, IFITM-1, IFITM-3, IFI16 and BST-2 differ between progressors and LTNPs. While no significant change was noted in the expression of the IFITM-1 (*p* = 0.57; Figure 4b), IFI-16 (*p* = 0.99; Figure 4d) and BST-2 (*p* = 0.11; Figure 4e) genes, a significantly reduced expression of the APOBEC-3G (*p* = 0.03; Figure 4a) and IFITM-3 (*p* = 0.002; Figure 4c) genes was noted in the progressors as compared to the LTNPs, indicating that the reduced antiviral activity could be partially involved in enhancing the course of HIV infection among the progressors.

### 3.4. TLR Stimulation of PBMCs Restores the Expression of Innate Cytokines and the Anti-Viral Host Restriction Factors in HIV-Infected Progressors

**Figure 5:**
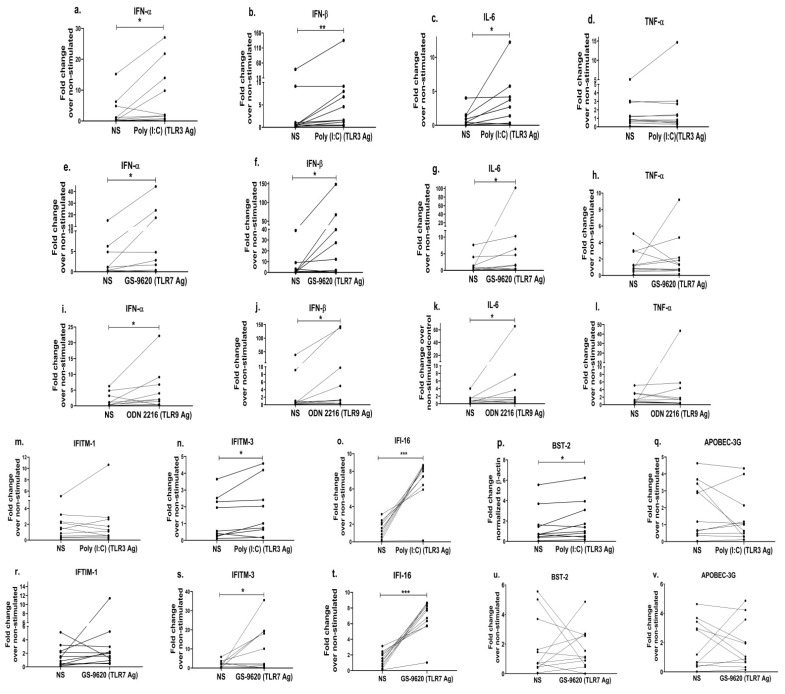
TLR stimulation in PBMCs restores the expression of innate cytokines and the anti-viral host restriction factors in HIV-infected progressors. PBMCs from progressors stimulated with Poly(I:C) (TLR3 agonist (1 uM)) were used to assess the gene expression of innate immune cytokines such as IFN-α (**a**), IFN-β (**b**), IL-6 (**c**) and TNF-α (**d**). Similarly, the gene expression analysis was performed by using PBMCs stimulated with GS-9620 (TLR7 agonist (1 uM)) (IFN-α (**e**), IFN-β (**f**), IL-6 (**g**), TNF-α (**h**)) and ODN 2216 (TLR9 agonist (3 uM))) IFN-α (**i**), IFN-β (**j**), IL-6 (**k**), TNF-α (**l**)), respectively. In addition, mRNA expression levels of host restriction factors such IFITM-1 (**m**), IFITM-3 (**n**), IFI-16 (**o**), BST-2 (**p**) and APOBEC-3G (**q**) in PBMCs stimulated with poly(I:C) (TLR3 agonists (1 uM)) and IFITM-1 (**r**), IFITM-3 (**s**), IFI-16 (**t**), BST-2 (**u**) and APOBEC-3G (**v**) in PBMCs stimulated with GS-9620 (TLR7 agonist (1 uM)) were quantified by RT-PCR. Fold change expression was calculated as 2^−ΔΔCt^ over non-stimulated control (NS). Statistical analysis was performed by using the Wilcoxon test. * indicates *p* value < 0.05, ** indicates *p* value < 0.01 and *** indicates *p* value < 0.001.

Since previous data [55,91] and ours indicate that TLR-mediated anti-viral effects/responses are impaired in the progressors, we next investigated whether the exogenous stimulation of the PBMCs of the progressors with a TLR agonist(s) would help improve the release of anti-viral cytokines. We found that the stimulation of PBMCs with Poly:(I:C) (TLR3 agonist) resulted in increased expressions of IFN-α (*p* = 0.04; 2.7 fold; Figure 5a), IFN-β (*p* = 0.006; 3.3 fold; Figure 5b) and IL-6 (*p* = 0.02; 3.5 fold; Figure 5c). Similarly, stimulation with GS-9620, a TLR7 agonist, showed a significant increase in the mRNA levels of IFN-α (*p* = 0.04; 3.3 fold; Figure 5e), IFN-β (*p* = 0.05; 5.5 fold; Figure 5f) and IL-6 (*p* = 0.03; 8.1 fold; Figure 5g) than that of the non-stimulated PBMCs. Stimulation with ODN 2216 (a TLR9 agonist) also resulted in increased expressions of the IFN-α (*p* = 0.04; 2.7 fold; Figure 5i), IFN-β (*p* = 0.02; 5.8 fold; Figure 5j) and IL-6 (*p* = 0.04; 8.5 fold; Figure 5k) mRNAs as compared to the non-stimulated cells. However, no significant change was noted in the expression of TNF-α post stimulation with the TLR3 agonist (*p* = 0.64; Figure 5d), TLR7 agonist (*p* = 0.57; Figure 5h) or TLR9 agonist (*p* = 0.83; Figure 5l).

We observed earlier in this study that progressors had a significantly reduced expression profile of type-1 IFNs (Figure 2d,e) and the exogenous stimulation using TLR agonists increased the expression of type-1 IFNs in the PBMCs of the progressors (Figure 5a,b,e,f,I,j). Further, given that the increased type-1 IFNs augment the downstream signalling pathways resulting in the production of ISGs [92], we therefore assessed whether the stimulation with TLR agonists subsequently could also improve the expression of anti-viral ISGs commonly identified as host restriction factors such as *IFITM-1, IFITM-3, APOBEC-3G, IFI-16* and *BST-2*/*tetherin*. We indeed noted that the stimulation of PBMCs with the TLR3 agonist increased the expression of the IFITM-3 gene by 1.3 fold (*p* = 0.04; Figure 5n), while the TLR7 agonist stimulation increased it to 5.4 fold (*p* = 0.05; Figure 5s), as compared to the unstimulated cells. Furthermore, we observed 5-fold increased expression of IFI-16 (*p* = 0.001; Figure 5o), a 1.3-fold higher expression of the BST-2 gene (*p* = 0.05; Figure 5p) after the TLR3 agonist stimulation and a 4.8-fold increased expression of IFI-16 (*p* = 0.001; Figure 5t) post stimulation with the TLR7 agonist as opposed to unstimulated cells. However, we did not notice any difference in the expression of the ISGs/anti-viral restriction factors in PBMCs post stimulation with the TLR9 agonist. Collectively, these data suggest that the stimulation of TLR-mediated signalling in the progressors not only ameliorates the innate immune response but also improves the host restriction activity, which could thereby help control HIV disease progression.

### 3.5. Inhibition of miR-155 Increases the Expression of Toll-like Receptors, Transcription Factors, Innate Immune Cytokines and Host Restriction Factors

**Figure 6:**
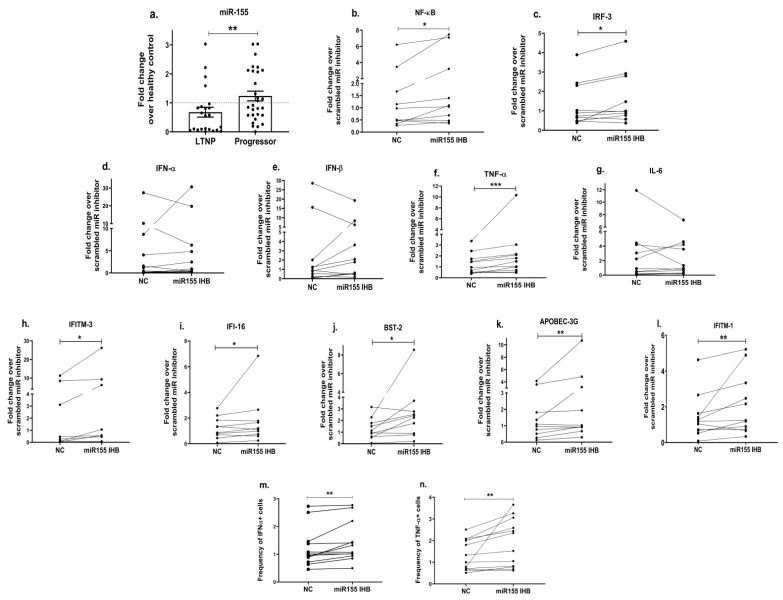
Inhibition of miR-155 increases the expression of transcription factors, innate immune cytokines and host restriction factors. MiR-155 (**a**) mRNA expression analysis was performed in HIV-infected study groups, i.e., LTNPs and progressors. Further, PBMCs of progressors transfected with miR-155 inhibitor or mock scrambled miR inhibitor control (denoted as NC) were used to examine the mRNA expression of genes encoding transcription factors such as NF-κB (**b**) and IRF3 (**c**), innate immune cytokines such as IFN-α (**d**), IFN-β (**e**), TNF-α (**f**) and IL-6 (**g**) and host restriction factors such as IFTIM-3 (**h**), IFI-16 (**i**), BST-2 (**j**), APOBEC-3G (**k**) and IFITM-1 (**l**). Fold change expression was calculated as 2^−ΔΔCt^ over control (NC) condition. Intracellular cytokine staining was performed to identify the frequency of IFN-α+ (**m**) and TNF-α+ (**n**) cells post transfection with miR-155 inhibition. Statistical analysis was performed using Wilcoxon test. * indicates *p* value < 0.05, ** indicates *p* value < 0.01 and *** indicates *p* value < 0.001.

We further wanted to understand the molecular mechanism involved in the regulation of innate immunity in the progressors. In addition to other protein factors, miRNAs have emerged as one of the important mediators of immunity [61]. MiR-155, in particular, has been shown to not only play a crucial role in regulating innate and antiviral responses in various viral infections [63] but has also been identified as a potential biomarker of HIV-induced immune dysfunction [65]. Therefore, we first examined the expression of miR-155 in the different cohorts of HIV-infected individuals—LTNPs and progressors. Interestingly, a significantly increased expression of miR-155 was noted in the PBMCs of the progressors as compared to the LTNPs (*p* = 0.009; Figure 6a). We further examined the specific role of miR-155 in regulating the innate events contributing towards HIV restriction by knocking down the expression of miR-155 in the PBMCs of the progressors. Since innate immune signalling triggers the activation of transcription factors such as nuclear factor kappa B (NF-κB) and the interferon regulatory factor (IRF), the mRNA expression of these transcription factors was assessed. We found that PBMCs transfected with the miR-155 inhibitor had increased expressions of TLR3 (2.1 fold, *p* = 0.03; Figure S2a), NF-κB (1.5 fold, *p* = 0.01; Figure 6b) and IRF-3 (1.2 fold, *p* = 0.04; Figure 6c) over the mock control (PBMCs transfected with scrambled miR inhibitor while TLR7 (*p* = 0.36; Figure S2b), and the TLR9 (*p* = 0.08; Figure S2c) gene expressions were not significantly different. Notably, we also found a 1.8-fold increased expression of TNF-α (*p* = 0.001; Figure 6f) post the transfection of PBMCs with the miR-155 inhibitor than that of the mock controls, while no significant difference was noted in the expression of type-1 interferons, i.e., IFN-α (*p* = 0.76; Figure 6d) and IFN-β (*p* = 0.7; Figure 6e) and IL-6 (*p* = 0.83; Figure 6g). We also assessed the intracellular expression of the TNF-α (pro-inflammatory) and IFN-α (type-1 IFN) proteins post transfection with miR-155 inhibition through flow cytometry. The frequency of cells expressing these cytokines was significantly increased in the miR-155-inhibited PBMCs compared to the mock/scrambled miR-inhibited cells (NC) (*p* < 0.003, Figure 6m,n).

Earlier in the present study, we noticed that the expression of host restriction factors was impaired among the progressors (Figure 4) and stimulating the TLR signalling resulted in an increased expression of some of these antiviral genes (Figure 5m–p); we therefore next questioned if miR-155 is also involved in modulating the expression of the antiviral host restriction factors. We found that miR-155 inhibition in the PBMCs indeed increased the expression of the *IFITM-3* gene by 1.9 fold (*p* = 0.02; Figure 6h), *IFI-16* by 1.4 fold (*p* = 0.05; Figure 6i), *BST-2* by 2 fold (*p* = 0.02; Figure 6j), the *APOBEC-3G* gene by 2.6 fold (*p* = 0.01; Figure 6k) and IFTIM-1 by 1.5 fold (*p* = 0.01; Figure 6l) in comparison with that of the mock cells.

As we noted that miR-155 inhibition increased the gene expression of TLR3, transcription factors (NF-κB and IRF-3), and host restriction factors (*APOBEC-3G, IFITM-3, IFI-16* and *BST-2*), we used an RNA hybrid prediction algorithm to examine the direct target sites for miR-155 on the genes of interest. For all the genes of interest, multiple miRNA–mRNA hybridization sites were identified. Based on the minimum free energy (mfe), the most optimum target sites for the genes were selected and are listed in Table 3. The observed binding targets of miR-155 for NF-κB, IRF-3, *APOBEC-3G, IFITM-3, IFI-16* and *BST-2*, as well as the TLR3 genes, thus indicate the regulatory role of miR-155 in the expression of these target genes. The inhibition of miR-155 resulted in an increase in the expression of several components involved in innate immune mechanism(s) such as TLR, transcription factors and host restriction factors in the PBMCs of the progressors. These findings collectively underline that miR-155 is one of the contributors associated with the impaired expression of anti-viral innate components among HIV-infected progressors.

## 4. Discussion

The innate immune mechanism(s) regulating disease progression during HIV immunopathogenesis is poorly understood, and a further understanding was attempted in this study. The expression profile of different TLRs and innate cytokines in PBMCs was identified during HIV infection. An increased expression of endosomal TLR genes, specifically TLR3, TLR7 and TLR9, was observed among HIV-infected individuals; however, no significant difference in the expression of TLR2 and TLR4 was noted when compared with the HIV-uninfected group. Additionally, the HIV-infected individuals had an increased expression of type-1 IFNs (IFN-α, IFN-β) and inflammatory cytokine (TNF, IL-6) genes. An increased expression of TLRs, namely TLR2, TLR3, TLR4, TLR7, TLR8 and TLR9, is well documented during HIV infection in various cells such as PBMCs [93], monocytes [37], monocyte-derived macrophages and mDCs [56] and gut epithelium [94]. Unlike other TLRs, an increase in TLR2 and TLR4 expression is more commonly reported in cells of myeloid origin, i.e., monocytes, MDMs and mDCs [37,56]. TLR signalling has been shown to promote antiviral activity through IFN-α/β release and proinflammatory cytokines during early stages of infection [95,96]. In corroboration with these reports, our findings also underline that TLR-mediated innate responses are altered during HIV immunopathogenesis.

The mounting of an early innate response is documented to be one of the crucial determinants influencing the disease outcome [97,98]. We next examined whether TLR-mediated responses differentially regulate HIV disease progression. To address this, the LTNPs were used as a model of slow disease progression *vis-a-vis* progressors. We observed that, compared to LTNPs, the progressors had reduced levels of the TLR3 and TLR9 genes as well as innate cytokines, viz. IFN-α, IFN-β and IL-6. A down-regulation of the TLR pathway and cytokines in progressors has been previously reported using a transcriptome analysis [55]. In another study, Scagnolari et al. observed that HIV-infected patients with high levels of HIV-1 RNA have reduced expressions of TLR-3, -7 and -9 compared to those with lower levels of viremia [91]. These findings indicate that TLR-mediated responses might have some role in controlling HIV growth and disease progression. Thus, we further intended to understand the dynamics between altered innate response and HIV-1 viral load and CD4+ T cell counts, which are the hallmarks of HIV disease progression [4,5]. We found that the HIV-infected individuals had an inverse association between HIV-1 viral load and CD4^+^ T cell count. Further, the progressors had a significantly higher VL and reduced CD4 count as compared to the LTNPs, as also previously reported [99,100,101]. Association studies have further shown that type-1 IFNs—IFN-α and IFN-β, were inversely correlated with viral load and positively correlated with CD4 count. While TNF-α had a positive association with CD4, no significant association was noted between IL-6 and CD4 count or HIV viral load. The role of the IFN-mediated response has been controversial in HIV immunopathogenesis. While increased type-1 IFNs have been associated with immune activation and CD4 T cell depletion [102,103], other studies highlight that the IFNs not only help in restricting post-virus entry [104], limiting the replication cycle [105], but also delay disease progression [106,107]. Our results are in agreement with later studies [105,106,107], suggesting that lower levels of type-1 IFNs are associated with disease progression. Given that the increased type-1 IFNs induce the expression of anti-viral host restriction factors that hamper HIV replication at multiple stages, subsequently resulting in controlled disease progression [90,108], and our findings indicate a differential expression of type-1 IFNs in the HIV-infected progressors, we next assessed the expression of various host restriction factors in the HIV-infected groups. It was found that the progressors had a reduced expression of *APOBEC-3G* and *IFITM-3* than that of the LTNPs, while the *IFITM-1* and *IFI-16* expression levels remained the same. A reduced expression of *APOBEC-3G* in progressors was also previously reported by Jin et. al. [109]. *APOBEC-3G* interferes with reverse transcription [110,111], while *IFITMs* have been shown to restrict HIV-1 entry and viral protein synthesis [112,113] and therefore might play a role in restricting viral growth and hence disease progression. Other restriction factors, such as *SLFN11, BST2* and *SAMHD1*, were reported to have a higher expression in LTNPs, indicating the role of ISGs/host restriction factors in virologic suppression [114]. Further, early-treated HIV-1-positive individuals showed a similar restriction factor expression profile to long-term non-progressors [114]. Taken together, our results indicate that impaired or compromised TLR-mediated innate responses contribute towards progressive HIV disease.

Since HIV infection results in immune dysregulation [2,3], we next attempted to explore whether stimulating TLR signalling through the use of specific agonists (Ag) would help improve anti-viral innate responses among the progressors. We found that PBMCs stimulated with TLR3 Ag (poly(I:C)), TLR7 Ag (GS-9620) and TLR9 Ag (ODN2216) increased the expression of type-1 IFNs (IFN-α, IFN-β) and IL-6. The addition of agonists to the intracellular TLRs, TLR7, TLR8 and TLR9, prior to infection in PBMCs has been shown to exhibit anti-HIV activity by inducing high levels of the type-1 interferons and interferon-stimulated genes [40]. Similarly, other in vitro and ex vivo studies reported that stimulation with the TLR3 (poly I:C) agonist in HIV-infected macrophages [41], the TLR7 agonist in HIV-infected monocytes [42], PBMCs and human macrophages [44], the TLR7/8 agonist in HIV-infected lymphoid tissue and PBMCs [43] and the TLR9 agonist in human lymphoid tissue [45] inhibited HIV replication and infection. All these studies highlighted that TLR-mediated immune responses are important in restricting HIV infection and/or increasing type-1 cytokines using in vitro or ex vivo infection models. However, here additionally, we report that stimulating TLR signalling in immunocompromised cells from progressors indeed helped in rescuing innate immune cytokine production. We further examined the effect of TLR agonists’ stimulation on the expression of host restriction factors. The stimulation of TLR3 increased the expressions of *IFITM-3* and *BST-2*, while TLR7 stimulation increased the expressions of the *IFITM-3* and *IFI-16* genes in the PBMCs of the progressors. TLR9 stimulation, however, did not result in an improved expression of the host restriction factors as compared to the non-stimulated PBMCs. Similar to our results, the stimulation of TLR signalling has been shown to increase the expression of various host restriction factors in different cells. While TLR3 stimulation with poly I:C increased the expression of *APOBEC-3G* and *Tetherin/BST-2* in HIV-infected macrophages [41] and the *BST-2* gene in activated PBMCs [115], stimulation with TLR7 Ag increased *BST-2, APOBEC-3G* expression in pDCs [116]. Contrary to our findings, TLR9 stimulation has been shown to induce the expression of *BST-2* and *APOBEC-3G* in the pDCs of HIV-infected individuals [116]. This could be due to the different cell types, i.e., PBMCs and DCs, used for the stimulation. TLR9 is highly expressed in pDCs [117,118], and in our study, the TLR9 agonist stimulation could not induce the expression of antiviral genes, which could be likely due to the low abundance of dendritic cells in PBMCs. These findings also indicate that each agonist has different levels of antiviral gene expression, and the degree to which each of these agonists contributed to the anti-HIV effect is still not known. However, it is likely that the stimulation of TLR signalling improves the overall antiviral state in the PBMCs of progressors, which might be helpful in controlling HIV disease progression. It would be interesting to investigate whether stimulating TLR signalling would also help in restricting virus growth/replication, which could not be accomplished in the current study. 

MiRNAs are one of the important regulators of innate immunity and are also shown to be involved in HIV immunopathogenesis [119,120,121]. Although the available reports suggest that miR-155 is involved during HIV infection [65,122], its role in regulating the components of innate immunity is hitherto not very clearly defined. Thus, we first assessed the expression of miR-155 in HIV-infected individuals with different disease courses and found an increased expression of miR-155 among the progressors as compared to the LTNPs. Previous studies among an Indian cohort have also shown that progressors had an increased expression of miR-155 than that of LTNPs [123]. Bignami et al., in another report, also documented an upregulated miR-155 expression in the CD4 cells HIV-1-infected patients compared to LTNPs [62]. Additionally, miR-155 has been shown to be associated with increased T cell activation, contributing towards immune dysfunction [65]. Taken together, our and previous findings envisage that upregulated miR-155 is associated with HIV immunopathogenesis and impaired immune functions. However, the role of miR-155 in the regulation of innate immunity and the molecular mechanism involved therein is not yet well known. Given that, in this study, an increased expression of miR-155 and a decreased anti-viral innate response were observed among the progressors. Additionally, though non-significant (*p* > 0.05), miR-155 levels were found to have an inverse trend of association with innate cytokines; we therefore examined whether the modulation of miR-155 would be helpful in restoring the innate immune components. Indeed, we found that inhibiting miR-155 expression in the PBMCs of the progressors resulted in significantly increased levels of transcription factors, NF-κB and IRF-3, TNF-α, as well as TLR3. miR-155 has been previously shown to inhibit IFN-β production by targeting TLR3 in avian macrophage cells [124]. MiR-155 also controlled IKK expression, which may lead to the downregulation of NF-κB, thereby forming a negative feedback loop [125,126]. We also noted that upon miR-155 inhibition, the expression levels of TLR7, TLR9, and type-1 IFNs-α/β were upregulated; however, they did not reach to the level of significance. Interestingly, we noted that upon stimulation with the TLR9 agonist, there was a significant decrease in miR-155 expression, while no change in miR-155 expression was noted with the TLR3 or TLR7 agonist stimulations (Supplementary Figure S4). This indicates the role of miR-155 in the regulation of the TLR-9-mediated innate axis, which needs further investigations. Furthermore, we found that miR-155 inhibition in the PBMCs of the progressors resulted in an increased expression of host restriction factors, namely *IFITM-1*, *IFITM-3*, *IFI-16* and *BST-2*. The miRNA–mRNA target prediction analysis further revealed that the transcription factors NF-κB and IRF-3, cytokines TNF-α and IFN-β and host restriction factors- *APOBEC-3G, IFI-16, IFITM-3* and *BST-2* are the binding targets of miR-155. These findings thus suggest that miR-155 has the potential to regulate the expression of host restriction factors. Similar findings were previously reported by our group, where we showed that miR-155 inhibition increased the expression of *APOBEC-3G, IFI-16* and *IFITM-3* in cervical epithelial cells [127]. The host restriction factors are the type-1 interferon-induced specialized host proteins that inhibit virus replication at multiple stages [112,128,129,130] and influence the course of HIV infection and disease outcome [131,132]. Previously, through polymorphism studies, the role of restriction factors such as *APOBEC-3G* [133], *BST-2* [134,135], *IFITM3* [136], etc., in association with disease progression has been described. Overall, we report that inhibiting miR-155 expression in the PBMCs of the progressors not only helped improve TLR3-mediated innate responses but also increased the expression of host restriction factors, which might be helpful in controlling the overall course of HIV disease progression. One of the limitations of our study is that we could not perform the TLR agonist stimulation and gene silencing experiments on the LTNP samples due to the unavailability of such individuals post-‘test and treat’ strategy. This could have helped us understand whether the treatments (with TLR or miR-155 inhibition) lead to only partial improvement or help to fully rescue the pathway. It would be of further interest to examine in future studies whether modulating miR-155 expression also helps in restricting virus growth and release among the progressors.

**Figure 7:**
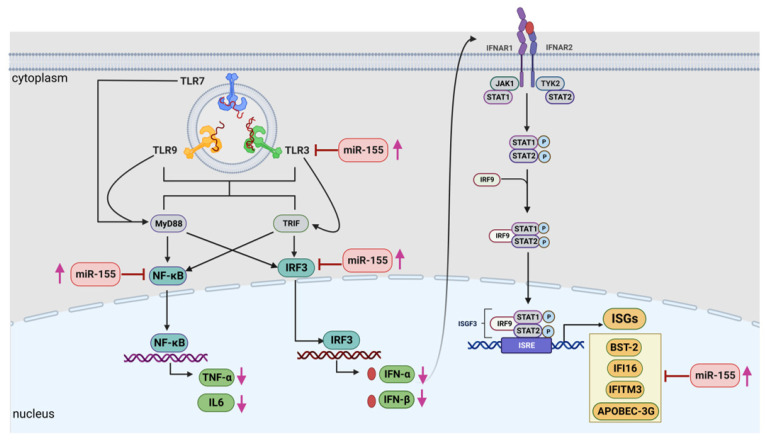
A graphical representation showing that miR-155 negatively regulates the anti-viral immune responses in progressors at multiple stages during the innate immune pathway. Increased expression of miR-155 in the PBMCs of progressors targets the expression of TLRs, in particular TLR3, transcription factors such as NF-κB and IRF-3, resulting in decreased expression of innate cytokines such as IFN-α, IFN-β. IL-6 and TNF-α, as well as host restriction factors viz. *APOBEC-3G, IFITM-3, IFI-16* and *BST-2*, which could result in rapid HIV disease progression in such individuals. Pink arrow denoted increased or decreased expression levels while red arrow indicate inhibition. This image is generated by using BioRender.

To conclude, we report that the stimulation of TLR signalling in progressors not only helped in alleviating innate cytokine expression but also improved the expression of anti-viral host restriction factors. Further, to the best of our knowledge, this is the first report that highlights the role of miR-155 in suppressing the innate immune environment among the progressors (Figure 7). The inhibition of miR-155 helped in rescuing the TLR-mediated anti-HIV cytokine expression and host restriction factors among the progressors, which might be helpful in controlling the disease progression. Further, since TLR-mediated innate responses improve adaptive immunity, it is likely that rescuing the innate response by modulating miR-155 would be helpful in promoting effector immune cells such as CD8 and NK cells, which needs further investigation. Improved innate and adaptive responses may also have implications in HIV latency, since innate immunity is one of the critical factors regulating the establishment of HIV latency early during infection. This study envisages that targeting miR-155 might be helpful in devising new strategic approaches to improve anti-HIV immune responses, subsequently leading towards controlled disease progression.

## Figures and Tables

**Table 1:** Genes with forward and reverse primer sequence.

Genes	Primer Sequences	Reference
β actin	F-5′-TCGTCCACCGCAAATGCTTCTAG-3′ R-5′-ACTGCTGTCACCTTCACCGTTCC-3′	[67]
NF-κB	F-5′- TCTCCCTGGTCACCAAGGAC-3’ R-5’- TCATAGAAGCCATCCCGGC-3′	[68]
IRF3	F-5′- ACCAGCCGTGGACCAAGAG-3′ R-5’-TACCAAGGCCCTGAGGCAC-3′	[69]
IFN-α	F-5′-ATTTCTGCTCTGACAACCTC-3′ R-5’- TGA CAGAGACTCCCCTGATG-3′	[70]
IFN-β	F-5′-GGTTACCTCCGAAACTGAAGA-3′ R-5’-CCTTTCATATGCAGTACATTAGCC-3′	[71]
TNF-α	F-5′- CTGGGGCCTACAGCTTTGAT-3′ R-5‘-GGCTCCGTGTCTCAAGGAAG-3′	[72]
IL-6	F-5′-ACCCCCAATAAATATAGGACTGGA-3′ R-5‘-GCTTCTCTTTCGTTCCCGGT-3′	[73]
IL-1β	F-5′-ATGCACCTGTACGATCACTG-3′ R-5’ -ACAAAGGACATGGAGAACACC-3’	[74]
TLR2	F-5′- CTTCACTCAGGAGCAGCAAGCA-3’ R-5’- ACACCAGTGCTGTCCTGTGACA-3’	[75]
TLR4	F-5′-GGTGCCTCCATTTCAGCTCT-3’ R-5′-ACTGCCAGGTCTGAGCAATC-3’	[76]
TLR3	F-5’- GCGCTAAAAAGTGAAGAACTGGAT-3’ R-5’- GCTGGACATTGTTCAGAAAGAGG-3’	[75]
TLR7	F-5′-GTTACCAGGGCAGCCAGTTC-3′ R-5’-ATGAGCCTCTGATGGGACAA-3′	[77]
TLR8	F-5′-TTTCAGAATAGCAGGCGTAA-3′ R-5′-AAGGGAAGATGTAAAGTCAGATAG-3′	[77]
TLR9	F-5′-GCATCTTCTTCCGCTCACTC-3 R-5′-TGTCCGACAGGTCCACGT-3’	[77]
IFITM1	F-5′-ACTAGTAGCCGCCCATAGCC-3′ R-5′-GCACGTGCACTTTATTGAATG-3′	[78]
IFITM3	F-5′-ATGAATCACACTGTCCAAACCTTCT-3′ R-5′-CTATCCATAGGCCTGGAAGATCAG-3′	[79]
IFI16	F-5′-ACTGAGTACAACAAAGCCATTTGA-3′ R-5′-TTGTGACATTGTCCTGTCCCCAC-3′	[80]
APOBEC-3G	F-5’-CGCAGCCTGTGTCAGAAAAG-3′ R-5’-CCAACAGTGCTGAAATTCGTCATA-3’	[81]
BST-2	F-5’-CCGTCCTGCTCGGCTTT-3′ R-5′-CCGCTCAGAACTGATGAGATCA- 3′	[82]

**Table 2:** Correlation analysis of innate cytokines with markers of disease progression, i.e., CD4 count and viral load among HIV-1-infected individuals.

Genes	CD4 Count (Cells/mm^3^)		Viral Load (Copies/mL)	
	*r*	*p*	*r*	*p*
IFN-α	0.53	0.0004	−0.42	0.01
IFN-β	0.45	0.0029	−0.47	0.005
TNF-α	0.37	0.0145	−0.22	ns

*r* = Spearman correlation coefficient; ns = non-significant.

**Table 3:** List of miR-155 binding targets on the 3′UTR regions of TLR3, transcription factors (NF-κB, IRF-3) and host restriction factors (APOBEC-3G, IFITM-3, IFI-16, BST-2) and their corresponding most optimal miRNA–mRNA duplex hybrid structures. The minimal free energy values (kcal/mol) of the miRNA–mRNA duplex are shown. miR-155 is shown in red and the target 3′ UTR of the genes are shown in green.

Gene	Position	Minimum Free Energy (mfe) (kcal/mol)	Binding Region	Most Optimal miRNA-mRNA Duplex Hybrid Structures
**TLR3**	2025	−24.8 kcal/mol	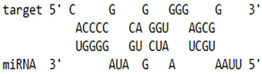	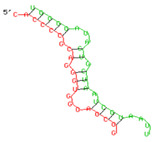
**NF-κB**	29	−20.5 kcal/mol	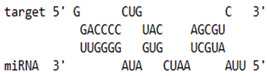	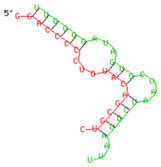
**IRF-3**	6	−19.4 kcal/mol	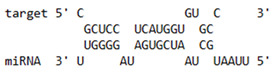	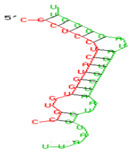
**APOBEC-3G**	6	−19.1 kcal/mol	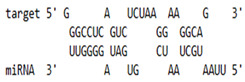	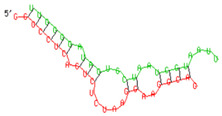
**IFITM-3**	101	−20.1 kcal/mol	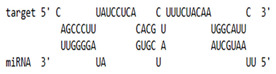	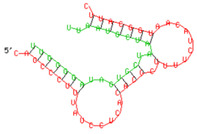
**IFI-16**	122	−19.2 kcal/mol	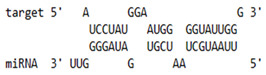	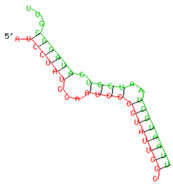
**BST-2**	136	−24.4 kcal/mol	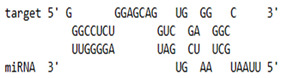	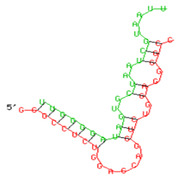

## Data Availability

Data are contained within the results section of this article.

## Supplementary Materials

The following are available online at https://www.mdpi.com/article/10.3390/v15112206/s1, Figure S1: Gating strategy to identify the intracellular expression of IFN-α and TNF-α proteins post miR-155 inhibition in the PBMCs of progressors. Figure S2: Validation of miR-155 depletion in PBMCs upon miR-155 inhibitor treatment. Figure S3: Inhibition of miR-155 increases the expression of TLRs. Figure S4: Effect of TLR stimulation on the expression of miR-155 in the PBMCs of progressors.
