# Supplementary material for: MiR-155 Negatively Regulates Anti-Viral Innate Responses among HIV-Infected Progressors
Source: Viruses. 2023 Nov 1;15(11):2206. doi: 10.3390/v15112206 (PMC10675553; doi:10.3390/v15112206)
Supplement: Supplementary file 1 [file viruses-15-02206-s001.zip › viruses-2625216-supplementary.pdf]

Supplementary Materials:

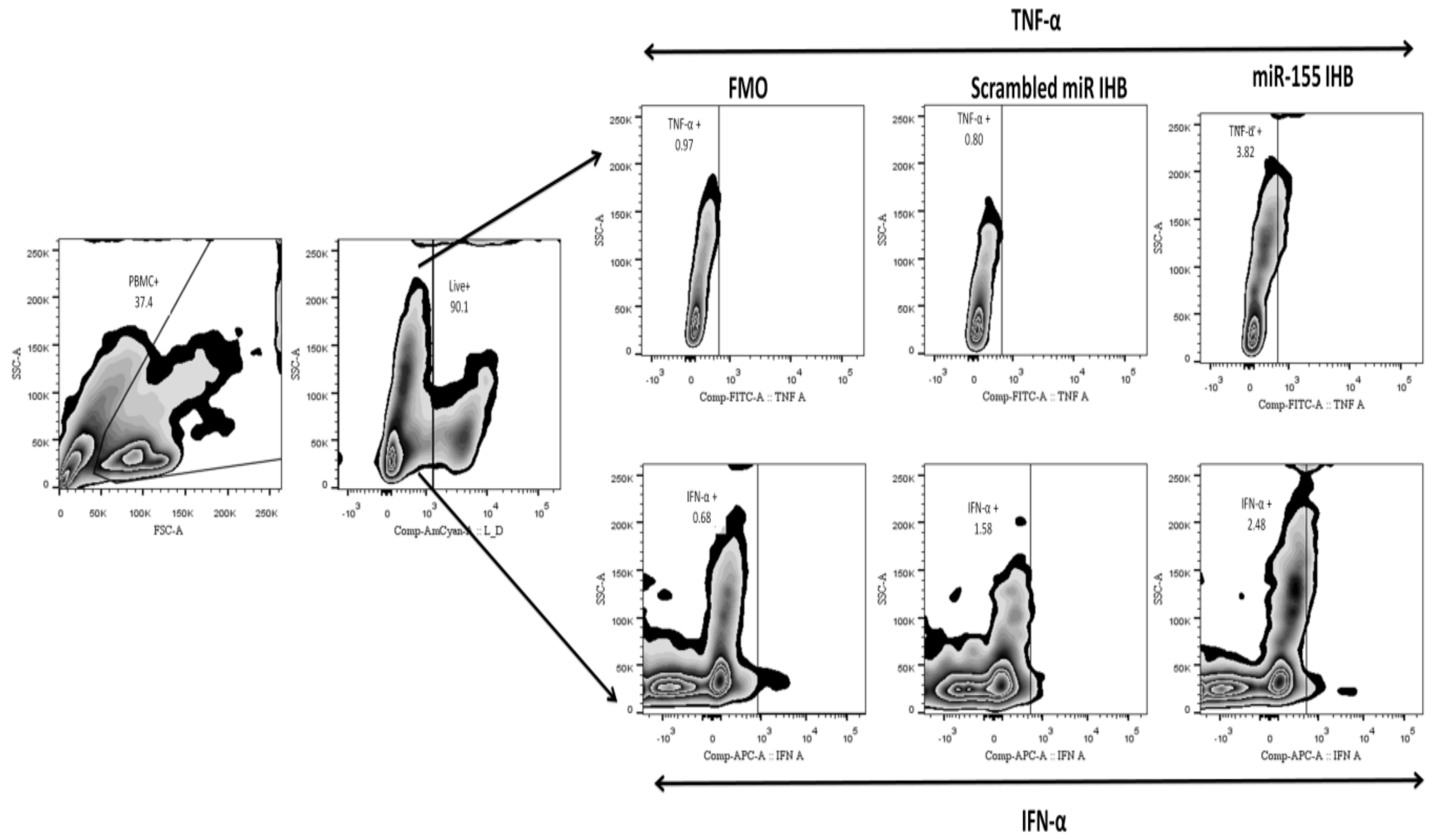

Figure S1: Gating strategy to identify the intracellular expression of IFN-α and TNF-α proteins post miR-155 inhibition in the PBMC of progressors. PBMCs population was first gated based on FSC and SSC plot followed by gating on live population. Live population were further gated to assess % population of IFN-α+/ TNF-α+ cells. FMO was used as control to gate these cytokines.

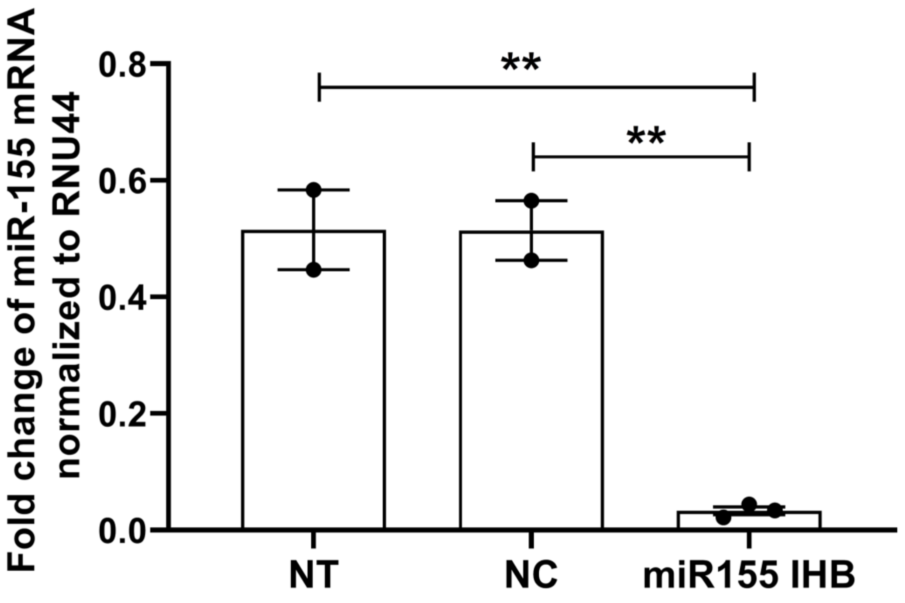

Figure S2: Validation of miR-155 depletion in PBMCs upon miR-155 inhibitor treatment. PBMCs were transfected with 200 nM of miR-155 inhibitor or mock scrambled miR inhibitor control (denoted as NC) or left untransfected (denoted as NT). After 24hrs, the expression of miRNA-155 gene was assessed by real-time PCR. Statistical analysis was performed by using Wilcoxon test. ** indicates p value < 0.01.

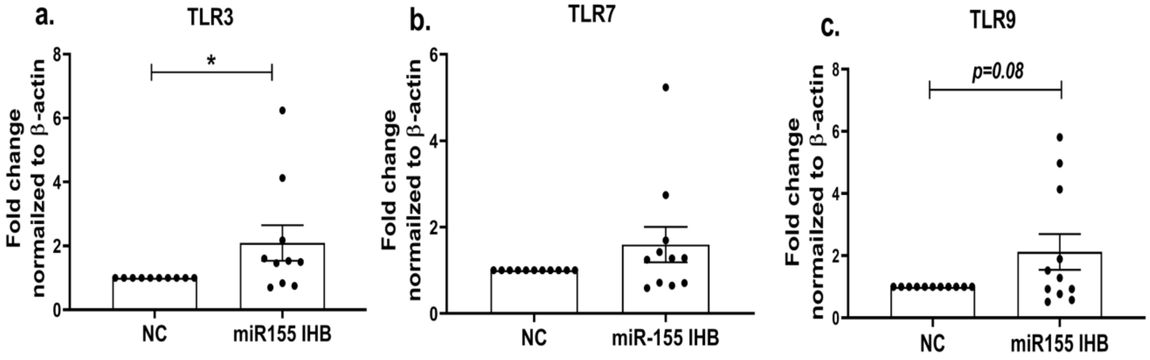

Figure S3: Inhibition of miR-155 increases the expression of TLRs. PBMCs of progressors transfected with miR-155 inhibitor or mock scrambled miR inhibitor control (denoted as NC) were used to examine the mRNA expression of TLR3 (a), TLR7 (b), TLR9 (c) by RT-PCR. Statistical analysis was performed by using Wilcoxon test. * indicates p value < 0.05.

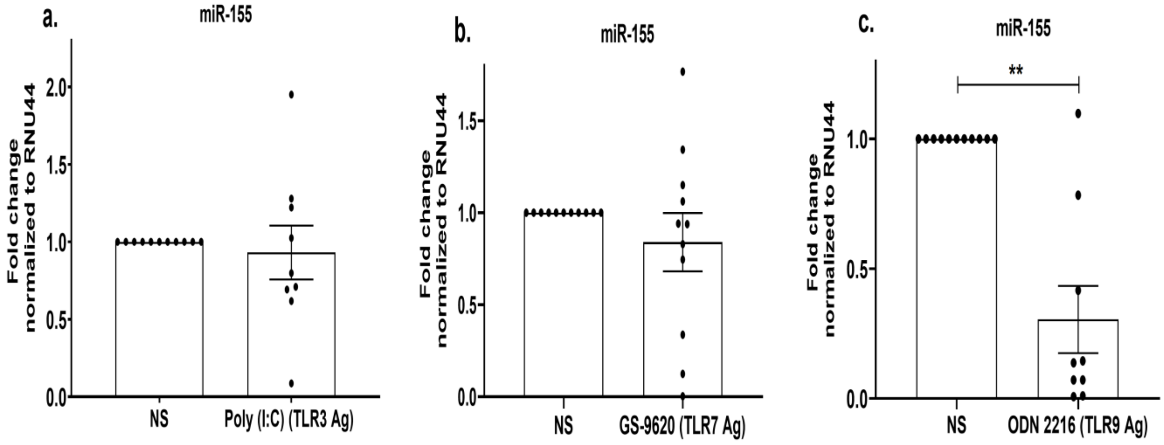

Figure S4: Effect of TLR stimulation on the the expression of miR155 in the PBMC of progressors. PBMCs of progressors were stimulated with Poly(I:C) [TLR3 agonist (1uM)] (a), GS-9620 [TLR7 agonist (1uM)] (b), ODN 2216 [TLR9 agonist (3uM)] (c) and gene expression of miR-155 was assessed by RT-PCR. Statistical analysis was performed by using Wilcoxon test. * indicates p value < 0.05.
